# An Intelligent EEG Classification Methodology Based on Sparse Representation Enhanced Deep Learning Networks

**DOI:** 10.3389/fnins.2020.00808

**Published:** 2020-09-30

**Authors:** Jing-Shan Huang, Yang Li, Bin-Qiang Chen, Chuang Lin, Bin Yao

**Affiliations:** ^1^School of Aerospace Engineering, Xiamen University, Xiamen, China; ^2^Shenzhen Institutes of Advanced Technology, Chinese Academy of Sciences, Shenzhen, China

**Keywords:** electroencephalogram, common spatial patterns, sparserepresentation, residual convolutional neural networks, fast compression

## Abstract

The classification of electroencephalogram (EEG) signals is of significant importance in brain–computer interface (BCI) systems. Aiming to achieve intelligent classification of EEG types with high accuracy, a classification methodology using sparse representation (SR) and fast compression residual convolutional neural networks (FCRes-CNNs) is proposed. In the proposed methodology, EEG waveforms of classes 1 and 2 are segmented into subsignals, and 140 experimental samples were achieved for each type of EEG signal. The common spatial patterns algorithm is used to obtain the features of the EEG signal. Subsequently, the redundant dictionary with sparse representation is constructed based on these features. Finally, the samples of the EEG types were imported into the FCRes-CNN model having fast down-sampling module and residual block structural units to be identified and classified. The datasets from BCI Competition 2005 (dataset IVa) and BCI Competition 2003 (dataset III) were used to test the performance of the proposed deep learning classifier. The classification experiments show that the recognition averaged accuracy of the proposed method is 98.82%. The experimental results show that the classification method provides better classification performance compared with sparse representation classification (SRC) method. The method can be applied successfully to BCI systems where the amount of data is large due to daily recording.

## Introduction

Brain–computer interface (BCI) is one of the research hotspots in the fields of biomedicine and signal processing in recent years. Brain–computer interface technology is a human–computer interaction method based on brain signals. It provides a communication channel for non-neuromuscular control. Brain–computer interface is a communication system that enables the human brain to interact with the external environment without relying on the peripheral nervous system and muscles.

In the BCI system, electroencephalography (EEG) signal is the manifestation of brain nerve electrical signals. It is also the basis of signal processing in the system. Electroencephalography signals comprehensively reflect the physical and chemical activities of the nervous system and are powerful tools for analyzing neural activity and brain conditions (Wu et al., 2008). Any changes in brain function and structure caused by neurological brain diseases can lead to abnormal brain electrical signals. In clinical medicine, the information processing of EEG signals not only provides an objective basis for the diagnosis of certain brain diseases, but also provides effective treatment for some brain diseases (Thornton, 2002). For a long time, doctors need to manually detect and analyze the waveform characteristics of EEG, with intensive labor and strong subjectivity. Therefore, the classification of EEG signals is of great significance to the identification, morbid prediction, and prevention of brain diseases.

In the BCI, the EEG signal is the main medium for human–computer interaction. An important part of the BCI system is processing the collected EEG signals to determine the type of commands issued by the brain. Motor imaging (MI) signal is a type of EEG signals. It refers to brain signals generated by imagining limb movement without actual limb movement. By analyzing the MI signal, it is possible to judge the imaginary’s movement intention and operate the external device. At present, the motion imaging control has great potential application value in various fields such as sports function rehabilitation, motor function assistance, and so on. Therefore, the MI signal becomes the most commonly used signal in the BCI. It is also the EEG signal studied in this article. Because of the non-stationarity of the EEG signal and the influence of a large number of background waveforms and artifacts, EEG classification is a challenging problem. At present, researchers have done a lot of work in various fields to study the feature extraction and classification of EEG signals.

Common spatial patterns (CSPs) is a popular method of extracting features in EEG studies. The CSP method has been applied successfully in many EEG classification studies (Ramoser et al., 2000; Grossewentrup and Buss, 2008; Mousavi et al., 2011; Keng et al., 2012). Other well-known feature extraction and dimension reduction methods such as principal component analysis and independent component analysis are also used frequently to improve the EEG classification accuracy (Ince et al., 2006; Guo et al., 2008; Talukdar et al., 2014). Autoregressive model and power spectral density estimation are also common feature extraction algorithms for EEG classification (Argunsah and Cetin, 2010; Seth et al., 2017). In the classification part, the frequently used classification methods include linear discriminant analysis (Rajaguru and Prabhakar, 2017), Bayesian method (Bashashati et al., 2016), BP neural network (Gao et al., 2012), support vector machine (SVM) (Liu et al., 2012), and so on (Wang et al., 2006; Yang et al., 2012; Roeva and Atanassova, 2016). The characteristics of EEG signals mainly include the following aspects: randomness, weakness, catastrophe, non-stationarity, low frequency, and non-linearity. Therefore, it is difficult to determine the representation and appropriate description (Gao et al., 2018).

Sparse representation is a fast developing field by constructing sparse linear models. It represents a given input signal as a linear superposition of base signals selected from a predetermined dictionary (Chen, 2016). It can find a suitable dictionary for ordinary densely expressed signal samples, and convert the samples into a suitable sparse expression form. Sparse representation can simplify learning tasks and reduce model complexity. Sparse representation has a large number of applications in the fields of signal acquisition, denoising, and image restoration (Elad and Aharon, 2006; Yang and Li, 2009; Li et al., 2013). The classification of EEG signals based on sparse representation is also developing gradually. Shin et al. (2015) proposed simple adaptive sparse representation-based classification (SRC) schemes for EEG classification, and the proposed adaptive schemes show relatively improved classification accuracy as compared to conventional methods without requiring additional computation. Zhou et al. (2012) proposed a method to learn a new dictionary with smaller size and more discriminative ability for the classification, and the experimental results of the EEG classification show that the proposed method outperforms the SRC method. Sreeja and Samanta (2020) proposed a weighted SRC (WSRC) for classifying MI signals to further boost the proficiency of SRC technique, and the experimental results substantiate that WSRC is more efficient and accurate than SRC.However, there is a contradiction between dictionary size and algorithm recognition accuracy.

Deep learning methodologies show outstanding performances in pattern recognition problems (Huang et al., 2019). Although the traditional pattern recognition method has been widely adopted, there is still a problem of relying on experience and prior knowledge in the process of manually selecting EEG signal features. In addition, feature extraction algorithms and feature classification algorithms use different objective functions so as to affect the pattern recognition accuracy. The deep learning neural network can extract more distinguishable and interpretable features of EEG signals. Meanwhile, the classification method based on deep learning neural network includes feature extraction and feature classification in a frame so as to avoid the loss of signal information caused by separating the two steps. Therefore, EEG classification based on deep learning related techniques has become a research hotspot. A deep belief network model (An et al., 2014)was applied for two class motor imagery (MI) classification, and the proposed model was shown more successful than the SVM method. Tsinalis et al. (2016) used convolutional neural networks (CNNs) to learn a single-channel EEG-based classification task filter for the automatic scoring of the sleep stage.Yang et al. (2015) used CNN to classify MI EEG signals. Chambon et al. (2018) proposed an end-to-end deep learning method to extract information from the EEG channel, which can finally correctly classify 91% of sleep stages from EEG signal. A recurrent CNN architecture was proposed by Bashivan et al. (2015) to model cognitive events from EEG data. A deep learning network with principal component–based covariate shift adaptation was proposed by Jirayucharoensak et al. (2014) for automatic emotion recognition.

Because the EEG signal contains a lot of noise and redundant information, it is not effective to obtain classification information directly from it. The sparse representation method can effectively remove the redundant information and retain the feature information that is beneficial to classification to best express the signal feature information. Meanwhile, the deep learning neural network has a wide range of applications in pattern recognition. Therefore, we combine the advantages of these two methods. We innovatively use the sparse features of the EEG signal as the input terminal of the deep neural network model and train the deep neural network model parameters to realize the automatic classification of the EEG signal.

In this article, we propose an intelligent EEG classification method based on sparse representation and enhanced deep learning networks. The features of the EEG signal are obtained through the CSP algorithm, and then the redundant dictionary with sparse representation is constructed based on these features. Subsequently, the sparse features were utilized as input of the fast compression deep learning networks to achieve the classification of EEG signals. The dataset downloaded from the website of BCI Competition 2005 (dataset IVa) and BCI Competition 2003 (dataset III) was used as the training and testing data. The classification results using the proposed method can reach an averaged accuracy of 98.82%.

The rest of this article is organized as follows. In *Methods*, we explain the methodology used for the EEG classification, including methodology overview, database and segmentation, and data preprocessing. We also explain the sparse representation classification model and the proposed fast compression deep learning networks. In *Results*, numerical evaluation and experimental results of EEG classification are shown, including evaluation metrics and the experimental classification results. Finally, we give the discussion and conclusion in *Discussion*.

## Methods

### Methodology Overview

**FIGURE 1:**
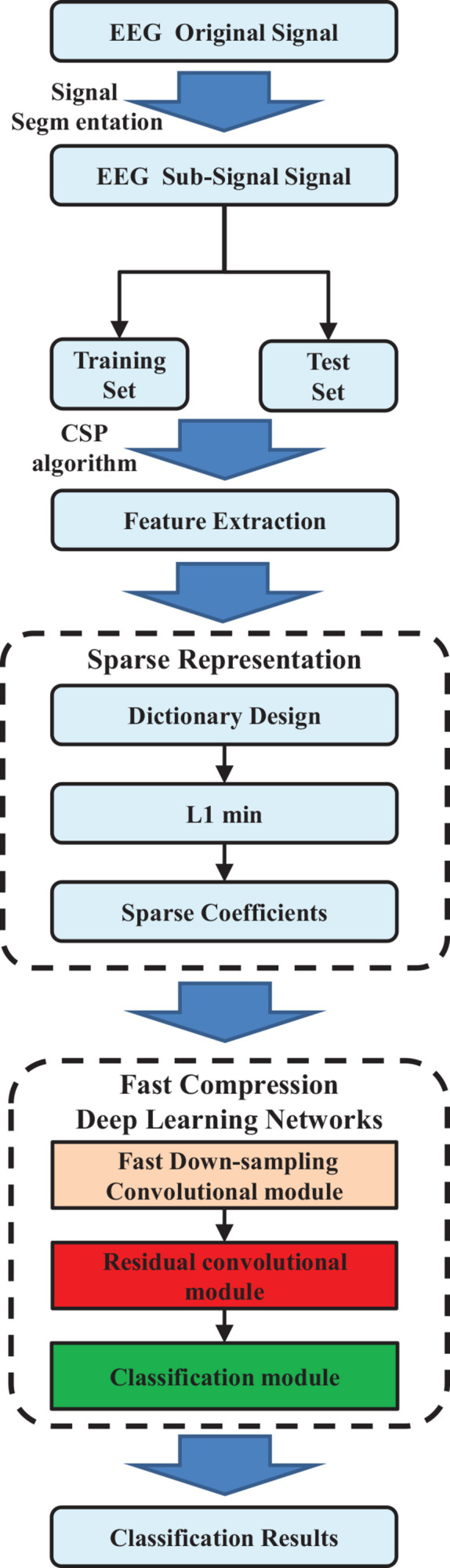
Overall procedures in EEG classification based on the proposed method.

The overall procedures of the proposed EEG classification method based on sparse representation and fast compression deep learning networks are shown in Figure 1. The original EEG signals were shared by the BCI Competition database (Blankertz et al., 2006). First, EEG waveforms are segmented into subsignals. Then the EEG signal features are obtained through the CSP algorithm, and the redundant dictionary with sparse representation is constructed based on these EEG signal features. Subsequently, the sparse features were utilized as input of the fast compression deep learning networks to complete the classification of EEG signals. Finally, EEG types are classified by the fast compression residual CNNs (FCRes-CNNs) classifier intelligently.

### Database and Segmentation

The experimental data in this article comes from the databases in BCI Competition 2005 (dataset IVa) and BCI Competition 2003 (dataset III). The databases contain datasets recorded by five different healthy subjects (aa, al, av, aw, ay). All five subjects underwent BCI experiments with three MI exercises of left hand, right hand, and right foot. In this experiment, only two types of right hand (R) and right foot (F) were used for data analysis, and they are named class 1 and class 2. Each EEG signal has 118channels. The common goal of BCI Competitions was to classify these MI tasks by using EEG signals recorded at C3, Cz, and C4 channels.First, the EEG signal is filtered by a bandpass filter of 0.05 to 200 Hz. Then, the EEG signal is digitized at a frequency of 1,000 Hz. Finally, the EEG signal is down-sampled to 100 Hz, and it is analyzed offline by the Berlin research team.

During the experiment, the subjects were seated in a comfortable chair, with their arms resting naturally on the armrests. At the beginning of the experiment, a visual cue in the form of a shoulder appeared in the center of the screen, informing the subjects of the MI task to be performed. The subject’s imagination time was 3.5 s. After the end of the MI, the subjects had a short time to rest and the rest time varied randomly from 1.75 to 2.25 s.

### Data Preprocessing

First, EEG waveforms need to be segmented to the 3-s time samples, and140 experimental samples can be achieved for each type of EEG signal. To reduce the interference from other sources such as electrooculogramsand electromyograms, 8- to 15-Hz bandpass filters were applied in this article (Gao et al., 2018). The CSP method is an effective method in the feature extraction problem of motion imaging signals. It is suitable for two classes (conditions) of multichannel EEG-baBCIs, so this article adopts the CSP method to filter the EEG signals and extract energy features. When the number of CSP filters is set as 32, after filtering operation, training and testing EEG samples can be converted to 32 CSP eigenvalues, which can be used for data classification.

### Sparse Representation Classification Model

Sparse representation represents a given input signal as a linear superposition of a small set of base signals selected from a predetermined dictionary. It can be said that the problem of sparse representation is a problem of representing a given input signal as simply as possible. For the EEG signals, a feature vector can be obtained by CSP (Legendre and Fortin, 1989):

(1) $${\text{asv}}_{i}=\left[{\text{v}}_{1\,i},{\text{v}}_{2\,i},\ldots ,{\text{v}}_{\mathrm{mi}}\right]\in {\text{R}}^{m}$$

where ***m*** is the sample dimension. If all the characteristic vector signals from different types of EEG signals are put in ***A*,** the matrix ***A*** can be written as the following form:

(2) $$\text{A}=\left[{\text{v}}_{1},{\text{v}}_{2},\ldots ,{\text{v}}_{n}\right]\in {\text{R}}^{m\times n}$$

Rajaguru and Prabhakar (2017) declared that if the training data from the **i^th^** category are enough, the test sample **y** from the same category can be shown as a linear combination of the training set associated with subject :

(3) $$\text{y}=\propto_{1}{\text{v}}_{1}+\propto_{2}{\text{v}}_{2}+\cdots +\propto_{n}{\text{v}}_{n}$$

where ∝ is the coefficient vector, and its elements are not all zero. By concatenating **A_i_**, the dictionary matrix A for all **k** classes can be acquired as **i** = **1**,**2**,…….**k**. The dictionary can be given as follows:

(4) $$\text{A}=\left[{\text{A}}_{1},{\text{A}}_{2},\ldots ,{\text{A}}_{k}\right]\in {\text{R}}^{m\times k}$$

**FIGURE 2:**
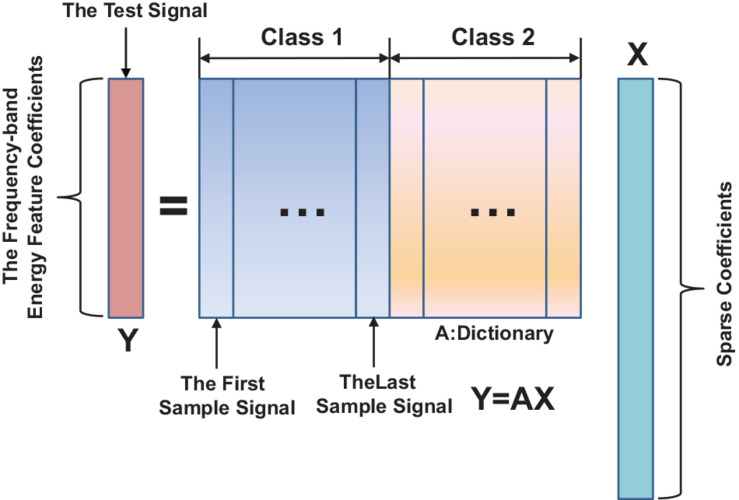
Sparse representation classification model.

If the EEG feature signal **y** is the tested signal, **y** can be written as a linear combination of.

all***nk*** training data.

$$\text{y}=\text{Ax}={\text{x}}_{1,1}\,{\text{v}}_{1,1}+{\text{x}}_{1,2}\,{\text{v}}_{1,2}+\cdots +$$

(5) $${\text{x}}_{1,n}\,{\text{v}}_{1,n}+{\text{x}}_{2,1}\,{\text{v}}_{2,1}+\cdots +{\text{x}}_{k,n}\,{\text{v}}_{k,n}$$

where **x** = [**v**_**1**,**1**_,**v**_**2**,**2**_,…,**v**_**k**,**n**_]**^T^** ∈ **R^nk^** are the coefficients vectors. In the ideal case,

**x** = [**0**,**0**…….,**v**_**i**,**1**_,**v**_**i**,**2**_,…,**0**,**0**…….,**0**] is a vector, which is mostly zero value except for those elements corresponding to the class of **i^th^**; thus, the corresponding class of EEG feature signals can be classified. The two types of sparse presentation classification operations are shown in Figure 2.

The test sample feature vector can be expressed as a linear combination of feature vectors of the training sample. The sparse coefficients ***x*** are used to encode the identity information of the test sample. The sparse coefficients are obtained by solving the linear equation of (5). Because the number of CSP filters is smaller than the number of training samples, the solution of ****x**** is not unique so that Eq.5 is underdetermined. New theories of sparse representation and compressed sensing have pointed out that L1 norm optimization can be used to solve underdetermined linear equations as long as ***x*** is sufficiently sparse. Based on the vector ***x*** and the test signal ***y***, L1-norm minimization equation can be listed as follows:

(6) x^=1arg min||x||1subjects.t.Ax=y

In the ideal case, when we obtained the estimate, it should have non-zero element corresponding to ***y***. Through analyzing the indices of the non-zero elements in ***${\hat{\text{x}}}_{1}$*,** the class of ***y*** can be determined. However, because of the modeling limitations and noise, ***${\hat{\text{x}}}_{1}$*** is not exactly zero but is close to zero. To resolve this problem, the following equation will be calculated generally as follows:

(7) $$\text{r1}\,\left(y\right)={||\text{y}-\text{A}\,\delta_{i}\,\left(x\right)||}_{2}$$

The test samples are classified according to the approximation residuals. The smaller the approximation residual, the closer the test sample is to this category. Therefore, the test sample is discriminated as the smallest category that approximates the residual. For each class *****i**,δ**_i_**(**x**)*** is obtained by nulling all the elements corresponding to the other class, then class ***i*** can be classified by analyzing the residuals, that is:

(8) $$\text{class}\,\left(y\right)=\text{arg}\,{\text{min}}_{i}\,{\text{r}}_{i}\,\left(y\right)$$

### Residual Neural Network Theory

Convolutional neural network is a special deep feedforward neural network designed by the inspiration of the concept of “receptive field” in the field of biological neuroscience (Liu, 2018). For traditional CNNs, the learning ability of the network will increase as the depth of the network increases. Meanwhile, the convergence speed of the network will slow down, and the time required for training will also become longer (Huang et al., 2020). The aim of residual networks is to address the degradation problem, which is defined as the decrease in accuracy as depth becomes greater than a certain threshold (He et al., 2016). Convolutional neural networks composed of residual block local deep neural networks units can address the degradation problem by facilitating the learning of identity mappings and solve difficulty in tuning of deep networks.

The residual neural network draws on the ideas of Highway networks (Srivastava et al., 2015). When the number of network layers reaches a certain threshold, the learning rate will decrease, and there is a risk of accuracy rate decline. The input of each layer in the general conventional CNN is derived from the output of the previous layer (Qin, 2019). It will be easily paralyzed if a network with many layers is performed with gradient calculations. The network structure of the residual network is similar to a “short circuit” structure. The output of the previous layers in the residual network does not go through the middle multiple network layers but directly serves as the input part of the network layer behind (Ji, 2019). Therefore, the residual structure has transformed the learning objectives. It no longer learns a complete mapping relationship from input to output, but the difference between the optimal solution **H**(**x**) and the input congruent mapping **x** (Huang et al., 2020). The residual calculation formula is as follows:

(9) F⁢(y)=H⁢(x)-x

The residual network can be regarded as a type of architecture consisting of a stack of residual blocks. The input data in the residual network come from different combinations of the previous network structure. This method introduces sufficient reference information to extract the effective features of the input EEG signal data (Liu, 2018). Because the paths in the network are relatively independent of each other, the regularity of the deep learning network structure is improved greatly.

### Proposed Convolutional Networks

**FIGURE 3:**
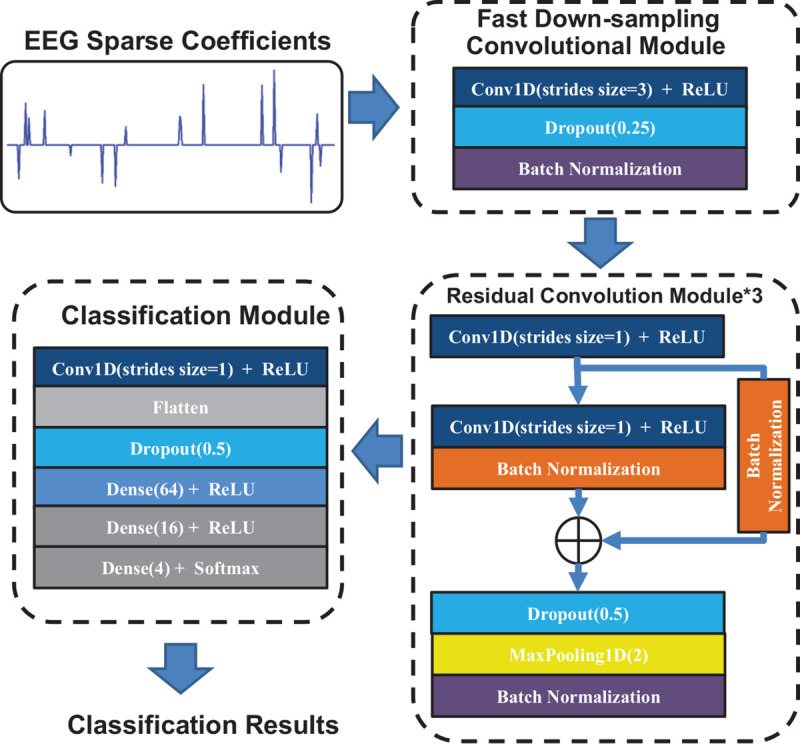
The architecture of the proposed FCRes-CNN.

In this section, we propose the FCRes-CNNs. As shown in Figure 3, the FCRes-CNN is mainly composed of a fast down-sampling module, three residual convolution modules, and a classification module. In the proposed FCRes-CNN model, the learning rate is set as 0.001, and the batch size parameter is set as 2,500.

In the proposed convolutional networks, convolutional layer with a stride of three is applied in the fast down-sampling module. Although a pooling layer also has effect of data compression, reducing overfitting, it will lose most of the original signal information while increasing network depth and spatial information loss due to the averaging nature of the pooling layer. Compared with the pooling layer, the convolutional layer with a large stride can adaptively learn the convolution kernel while compressing the input data (Huang et al., 2020). Therefore, we applied a convolutional layer with a large stride instead of a pooling layer in the fast down-sampling module.

The fast down-sampling module consists of a convolutional layer, a random dropout layer, and a batch-normalization layer. The convolutional layer with a stride of 3 is the main part of the fast down-sampling module. A random dropout layer and a batch-normalization layer follow the convolutional layer to enhance the generalization of the networks model. The fast down-sampling module can effectivelysimplify the calculation of deep network models, reduce data redundancy, and promote model learning (Huang et al., 2020).

Convolutional layers in series are applied in the residual convolution module, which are followed by residual short circuit. Then, a random dropout layer is added after the convolutional layer, and the max-pooling layer is applied to down-sample the EEG signal feature vectors.

In the classification module, a convolution layer is first used to reduce the dimension of the feature vectors. Then, a flattened layer follows the convolution layer. After the flattened layer, a random dropout layer is applied to prevent overfitting.

## Results

### Evaluation Metrics

The accuracy and loss were used as the evaluation criteria in the pattern recognition field.Therefore, we used the two evaluation criteria for the classification performance of EEG types. The accuracy and loss were calculated through Eqs10 and 11.

(10) $$\mathrm{Accuracy}\left(\%\right)=\frac{\mathrm{TP}+\mathrm{TN}}{\mathrm{TP}+\mathrm{TN}+\mathrm{FP}+\mathrm{FN}}\times 100$$

where *TP* stands for true positive, meaning the correct classification as class 1 of EEG; *TN* stands for true negative, meaning correct classification as class 2 of EEG; *FP* stands for false positive, meaning incorrect classification as class 1 of EEG; and *FN* represents false negative, meaning incorrect classification as class 2of EEG (Yin et al., 2016).

As for the metric of loss, it is defined as the difference between the predicted value of the EEG classification model and the true value for aspecific EEG sample (Huang et al., 2020). In this study, the mathematical expressionof categorical cross entropy loss is shown as Eq.11.

(11) $$\mathrm{loss}=-\frac{1}{\text{n}}\,\sum_{i=1}^{n}{\hat{y}}_{\mathrm{i1}}\,{\text{lny}}_{\mathrm{i1}}+{\hat{y}}_{\mathrm{i2}}\,{\text{lny}}_{\mathrm{i2}}+\cdots +{\hat{y}}_{\mathrm{im}}\,{\text{lny}}_{\mathrm{im}}$$

where **n** represents the number of EEGsamples; **m** represents the number of EEG types; $\hat{\text{y}}$ represents the predictive output value; and **y** represents the actual value.

### The Experimental Classification Results

In classification for the EEG signals of classes 1 and 2, each class can get 140 groups of 32 eigenvalues after the above data processing. Based on the training samples, the redundant dictionaries of sparse classification algorithm were constructed by using the CSP eigenvalues obtained by classes 1 and 2. Then, we scrambled all the EEG training sample data randomly and then selected the last 100 samples as the testing set. This approach ensures that the distribution of the training set and testing set is random and uncertain, and it can better reflect the classification effect of the proposed classifier.

The classification of EEG signals was completed based on the classification algorithm described insection “Methods.” The raw EEG waveforms are segmented into subsignals. The features of the EEG signal are obtained through the CSP algorithm. Then the redundant dictionary with sparse representation is constructed based on these features. Finally, the sparse features were utilized as input of the fast compression deep learning networks to complete the classification of EEG signals. The experiment runs on a PC with 16GB of memory and 16GB of GPU memory.

**FIGURE 4:**
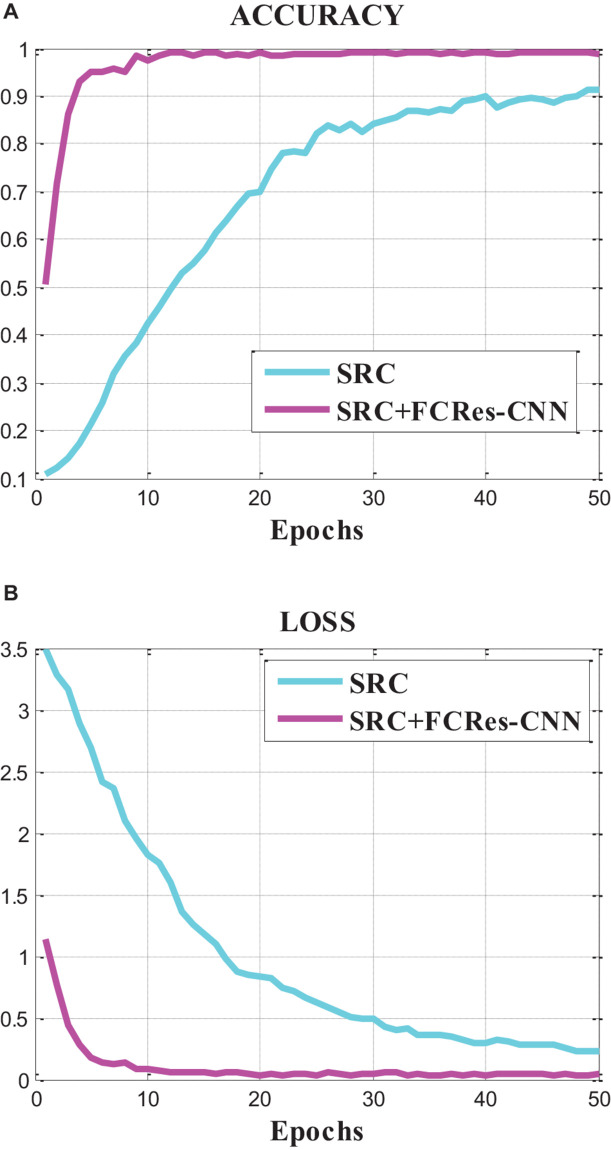
Accuracy and loss curves of the SRC and the proposed method.

Figure 4 represents the accuracy and loss curves of the sparse representation algorithm (SRC) and the proposed classification method (SRC +FCRes-CNN). From Figure 4, we can find that the accuracy value curve convergence rate of the proposed model is faster than that of SRC model, and the final accuracy convergence value of the proposed model is also much higher than that of SRC model. The loss value curve convergence rate of the proposed model is faster than that of SRC model, and the final loss convergence value of the proposed model is also much lower than that of SRC model. From these results, we can conclude that the proposed model achieves a higher average accuracy with lower loss than the SRC model based on the classification results of EEG signals. The proposed model outperforms the SRC model in the EEG classification application.

In the contrast experiment, the SRC model achieved an average accuracy of 88.79% and an average loss of 18.10%. In contrast, the proposed SRC +FCRes-CNN model achieved an average accuracy of 98.82% and an average loss of 4.74%. In this article, the total number of EEG training trials is 280. For the training process of deep learning, the number of samples is still insufficient. If the number of training samples is sufficient, the accuracy of classification will be further improved.

## Discussion

In this article, we proposed an EEG classification method based on sparse representation enhanced deep learning networks.

The original EEG signals were shared by the BCI Competition database. In the procedure of the proposed method, EEG waveforms of classes 1 and 2 are segmented into subsignals. The 3-s time samples after the prompt to conduct the classification experiment was applied, and 140 experimental samples can be achieved for each type of EEG signal. The CSP algorithm is used to obtain the features of the EEG signal. Then the redundant dictionary with sparse representation is constructed based on these features. Finally, the sparse features were utilized as input of the fast compression deep learning networks to complete the classification of EEG signal. The EEG classification is performed in the FCRes-CNN classifier automatically and intelligently.

The accuracy result of the proposed method on BCI Competition dataset Iva and dataset III is 98.82%, which is higher than the sparse representation classification method. The proposed method performs higher classification accuracy than other methods in literature by a training even using only a few samples, which is 280 trials in this article. We believe that the proposed method is of great significance for BCI applications that require real-time EEG classification of daily life use.

## Data Availability Statement

Publicly available datasets were analyzed in this study. This data can be found here: http://www.bbci.de/competition/iii/, http://www.bbci.de/competition/ii/#datasets.

## Author Contributions

J-SH, B-QC, and BY conceived and designed the classification method. CL and YL performed the experiment. J-SH preprocess and analyzed the data and wrote the manuscript. BY and B-QC reviewed and edited the manuscript. All authors read and approved the manuscript.

## Conflict of Interest

The authors declare that the research was conducted in the absence of any commercial or financial relationships that could be construed as a potential conflict of interest.
